# On the Pathology of Spontaneous Hæmorrhage

**Published:** 1852-06

**Authors:** A. B. Palmer


THE NORTH-WESTERN

MEDICAL AND SURGICAL JOURNAL.

NEW SERIES.

VOL. I. JUNE, 1852. No. 2.

### ORIGINAL COMMUNICATIONS.

ARTICLE I.—*On the Pathology of Spontaneous Hæmorrhage.* An Essay read before the Chicago Medical Society, February, 1852, by A. B. PALMER, M.D.

THERE are few subjects in Pathology more involved in obscurity —at least in regard to which there are more obscure points, or upon which the writings of our standard authors are less satisfactory, than that of the proximate cause, or essential nature of the hæmorrhagic condition. The vagueness and apparent contradictions of the books on this subject, arise, no doubt, in part from the fact, that, contrary to what the terms of the proposition for this evening's consideration* would seem to indicate, the pathological character of the hæmorrhagic tendency or state is *not always the same.* There is no one condition of the solids, of the fluids, or of the vital forces which is always present. There is no one circumstance, excepting the feebleness of the forces which retain the blood within its proper channels, compared to those which tend to its escape, producing the actual flow, which is a necessary attendant upon the disease.

This being the case, the proper consideration of the pathology of hæmorrhage will embrace a reference to the various conditions

* The subject previously appointed for the evening was "The essen[illegible]al nature of the Hæmorrhagic state."

which are known or supposed to exist in the disease, and to the numerous causes which may contribute to the production of the symptoms which mark it.

A complete treatise on this subject, embracing a full consideration of all the points involved, would extend far beyond the limits imposed by the present occasion, and of course will not be expected. I shall attempt, however, what I have not seen accomplished, at least in the same manner, in any of the books, viz.: to give system and form to the subject,—to place it in a position more favorable for investigation,—to indicate the mode in which the investigation should be conducted, rather than to fully investigate it myself.

The proximate cause of an escape of blood from its natural situation in the vessels, must consist in an *abnormal* condition either of

1st, The structure or physical qualities of the vessels from which it escapes;

2d, In an abnormal condition of the quantity or quality of the blood itself; or,

3d, Of the forces with which the blood is propelled.

One or more of these conditions must be present in all hæmorrhages. A derangement in the texture of the vessels, as a solution of their continuity, from whatever cause may be sufficient to produce the bleeding, while the condition of the blood and the force with which it circulates remain natural. Or, on the other hand, the condition of the blood may be such that though the texture of the vessels is healthy, and the force of the circulation normal, it may pass out. Or, again, the force with which the blood is impelled may be such, that though it be in a healthy condition and the tissues in a previous state of integrity and health, hæmorrhage may be the result. But although either one of these conditions is sufficient of itself to produce the phenomena, yet usually two or more are combined, when hæmorrhage actually occurs.

We shall be able to obtain a more clear impression of the subject by considering first these causes of hæmorrhage separately, and afterward in some of their combinations.

I. We will first consider the morbid conditions of the physical structure of the blood vessels which predispose, or give rise, to hæmorrhage.

These morbid conditions of the structure of the blood vessels in hæmorrhage are various, and I shall enumerate them in the order in which they may occur to me.

One abnormal condition of the blood vessels, disposing their contents to escape from them, consists in a *variation from their natural size* or circulating capacity,—and particularly a disproportion in these respects between the arteries and veins.

An equal enlargement of both the arteries and veins of a part, will lead to an increased quantity of blood in that part, and in a degree dispose to its effusion; but if the larger arterial branches leading to a part, or the capillary arteries distributed within it, become preternaturally enlarged while the veins retain their natural size,—or, if the veins within, or leading from a part, are contracted, pressed upon, or their circulating capacity in any manner diminished, while the arteries are normal,—or, if from any cause, or in any manner, the arteries are proportionably larger than the veins—their circulating capacities greater, the tendency to hæmorrhage is farther increased; indeed congestion is a necessary consequence, and hæmorrhage a very probable one.

As examples of changes in the circulating capacities of the larger veins leading from organs obstructing the returning circulation through them, and producing hæmorrhage, may be mentioned, the mechanical pressure of a diseased liver upon the walls of the portal vessels, causing hæmatemesis, melaena, and hæmorrhoids —the pressure of the gravid uterus upon the iliac veins, producing rupture and hæmorrhage of the veins of the lower extremity—the pressure of tuberculated lungs upon the pulmonary veins, producing some cases of hæmoptysis, &c., and also may be mentioned impairment of the valves of veins, or varicose dilatation from want of tonicity, as in other cases of hæmorrhoids, and rupture of vessels in the lower extremities.

As instances of hæmorrhage from the undue development of arteries, those from some forms of næva materni, or arterial anastomosing aneurisms, are most striking.

All these cases are specimens of structural changes affecting the circulating capacity of the vessels, but not the integrity of their cohesive force. But an alteration in the *texture* of their walls—a structural change in their *tissue* constitutes a cause of hæmorrhage.

This alteration of the texture of vessels, leading to tenderness, rupture, and hæmorrhage, may be produced by the deposition of arethomatous, scrofulous, ossific, or (perhaps) typhoid matter, within their coats,—or may consist in softening from inflammation, cachexy, or other forms of malnutrition,—aneurismal dilatation, or whatever degeneration, producing a feebleness of cohesive force,—or the alteration of texture may consist in actual *destruction* of portions of the vessels, as from mechanical violence, chemical reagents, and the process of ulceration.

Examples of hæmorrhage from the various deposits within the coats of blood vessels, are found in the rupture of large vessels and the spontaneous formation of false aneurisms, at last completely yielding. And examples of those from softening, produced by inflammation, cachexy, &c., are found occurring during the later stages of the progress of these diseases.

Hæmorrhages arising from destruction of the continuity of vessels by mechanical violence or chemical agents, do not belong to our present subject (spontaneous hæmorrhages), but those arising from lesions produced by ulcerations are included. Of these may be mentioned as specimens, hæmoptysis, from tuberculous ulceration of the lungs in the latter stages of consumption,—hæmorrhages from cancerous ulcerations in different locations,—hæmorrhages from mercurial ulcerations, as from the mouth in poisoning from that metal,—from gangrenous ulceration, as in cancrum oris,—from phagedenic ulceration, as in some cases of syphilis,—from aphthous ulceration, as in bleeding from various parts of the mucous membrane in that disease,—hæmorrhage from scorbutic ulcerations, as of the gums in scurvy—and hæmorrhage from typhoid ulceration, as in melaena in typhus and typhoid fevers.

But besides the more palpable alterations of the tissues of blood vessels, producing hæmorrhage, to which we have alluded, it is alleged that such other changes may occur as to give rise to sanguineous effusions by a morbid relaxation of fibres and dilatation of the natural pores without any positive rupture.

That firm and normal blood disks are incapable of passing through the unbroken walls of healthy vessels, must be evident to any one who has looked at these disks through the microscope in their natural channels. Indeed, though the blood disks are seen under glasses, of comparatively large size, no pores whatever are discernible in the walls of the vessels. Still, that thin fluids pass through these walls when in the natural state is unquestionable, proving their porosity,—and when their tissues are weakened and relaxed, as in low fevers and other forms of debility, it is possible that the blood globules, especially if they should be changed in their firmness and consistence, may be pressed through without any real solution of continuity. At any rate, the fact is unquestionable that sanguineous fluid has been poured out in considerable quantities from various mucous surfaces and even from the skin, without any discernible breach of vessels or of the surface.

In these cases, though the blood is usually at fault, as we shall shortly see, yet the texture of the vessels may be so changed as to allow particles to pass through them that would not, were they firm and natural; and the doctrine is held by some highly respectable authorities, that blood may, and often does, pass out of the vessels which contain it and escape from the body, without any actual solution of continuity. On the other hand there are others, and among them Vogel, of more recent and perhaps higher authority, who treat, as entirely untenable, the idea that undissolved blood corpuscles *ever* pass through the unbroken walls of blood vessels, however relaxed. But whether modified blood disks ever pass through the natural though relaxed pores of vessels or not, in hæmorrhages from the capillaries, these vessels are often in fault, possessing less than their natural firmness and consistence.

II. In the second place, we are to consider the condition of the blood itself, its quantity and quality, as predisposing to hæmorrhage.

And 1st, the blood may be excessive in quantity, constituting plethora.

This condition disposes to hæmorrhage from the over-distention, and consequent increased pressure, from within, upon the vessels. The simple mechanical principle upon which such a result depends, is too obvious to require illustration.

Over fullness of the blood vessels may be attended either with increased or diminished strength and irritability of the moving fibre, producing the two forms of *sthenic* and *asthenic* plethora. These conditions differ in many respects, but both tend to the production of hæmorrhage,—the former of a more active, and the latter of a more passive kind. In this connexion, reference is had solely to the effect which an increased quantity or bulk of the circulating fluid produces by its increased mechanical pressure,—none is had to an increased proportion of hæmatin or other nutrient qualities of the blood. The whole mass may be poor in quality—thin and watery—the patient actually more or less anæmic, while the quantity of the circulating fluid is large.

2d, The blood may be changed in *quality* so as to dispose to hæmorrhage.

One of the most frequent of the changes tending to this result, is a *deficiency* of *fibrin* relative to the amount of *red corpuscles.*

There are two conditions of the blood in which this disproportion exists. In one, the proportion of fibrin to the whole mass of blood is the same as in health; but there is an increased proportion, a decided excess, of red corpuscles. This condition exists in sthenic plethora, accompanied by a florid complexion and active circulation, and tends to the production of active hæmorrhages, apoplexy, &c.—In the other condition there is a positive diminution of fibrin, while the globules remain in their usual proportion or may be even diminished, as in the low forms of fevers, and the like, and the hæmorrhages occurring in these forms of disease not accompanied by ulcerations or other organic lesions, are usually dependent, in a great degree at least, upon this cause. In this condition of the blood it does not readily or firmly coagulate, and when any local lesion occurs, or blood begins from whatever cause to flow, the discharge is apt to be profuse.

Another supposed change in the quality of the blood, producing other effects in the fluid, favorable to hæmorrhage, is a superabundance of alkali—usually ammonia. Observations have not been sufficiently numerous to fully establish this point, but some few analyses of Simon, and I believe others, render it highly probable. Alkalies certainly possess more or less solvent effects upon the solid constituents of the blood.

The breaking down of the blood disks and the solution of the hæmatin or coloring matter in the serum—whether that breaking down be caused by the action of chemical agents upon the membrane enclosing the hæmatin, or whether the membrane yields from a failure in its vital tonicity,—is another change in the quality of the blood strongly disposing to hæmorrhages. In this possible, and actual, though rare condition of the blood in life, we can easily understand how effusion may take place through the natural pores of the vessels without rupture of tissue; but this state does not occur excepting as the result of poisons, in connection with gangrene, or in putrid and petechial fevers, generally marking a fatal termination.

It is believed that in some cases changes in the quality of the fibrin and red particles take place, disposing to hæmorrhage, short of their solution, and where these constituents remain in their normal proportions. This position is rather a subject of plausible conjecture than of ascertained fact, or unquestionable inference; but the cases of purpura and scurvy seem to favor it. In purpura there is no material deviation from the natural standard of the relative proportions of the fibrin or globules, and certainly neither of these elements is in a state of solution, and still the extravasation of the blood is supposed to depend, in part at least, upon some abnormal condition of that fluid; and in scurvy, the fibrin—the coagulable material and the element which is regarded as the one arresting hæmorrhage—is actually in excess, while the proportions of the other elements are nearly normal; and yet here, too, the hæmorrhage is regarded as depending, in part, upon some improper condition of the blood. But the fault in these cases may be entirely, as we know it is in part, in the vessels.

A general impoverished condition of the blood, in which there is a deficiency of the globules, the albuminous and fibrinous constituents, and in which its vitality also is defective, contributes strongly to a hæmorrhagic tendency; both in consequence of its being more fluid in its consistence—thereby passing more readily through the tissues, and of its reacting, in this condition, less efficiently upon the solid fibres, thereby inducing debility and relaxation.

[TO BE CONTINUED.]

